# Novel Astroviruses in Children, Egypt

**DOI:** 10.3201/eid1712.110909

**Published:** 2011-12

**Authors:** Salwa F. Ahmed, Peter J. Sebeny, John D. Klena, Guillermo Pimentel, Adel Mansour, Amel M. Naguib, Jody Bruton, Sylvia Y.N. Young, Lori R. Holtz, David Wang

**Affiliations:** US Naval Medical Research Unit No.3, Cairo, Egypt (S.F. Ahmed, P.J. Sebeny, J.D. Klena, G. Pimentel, A. Mansour, J. Bruton, S.Y.N. Young);; Ministry of Health, Cairo (A.M. Naguib);; Washington University School of Medicine, St. Louis, Missouri, USA (L.R. Holtz, D. Wang)

**Keywords:** Novel astrovirus MLB1, VA2, Egypti, children, stool, diarrhea, Middle East

**To the Editor:** Human astroviruses (HAstVs) are a common cause of gastroenteritis in children, the elderly, and immunocompromised persons (*1*). Up to 10% of acute viral gastroenteritis in children and 0.5%–15% of diarrheal outbreaks are attributed to astroviruses (*2*). Until 2008, eight classical astrovirus serotypes were known to cause human disease; in Egypt, HAstV-1 is the most frequent astrovirus serotype detected (*3*). Recently, 5 novel astroviruses have been discovered in human fecal samples from patients with diarrhea or acute flaccid paralysis (*4**–**7*). Because the prevalence of these virsues in the Middle East is unknown, we screened fecal samples from children with diarrhea residing in Egypt to ascertain the prevalence and diversity of these novel astroviruses.

Fecal samples were collected from a cohort of 364 symptomatic children <5 years of age who had diarrhea and were seeking medical care at Abu Homos Hospital in the Nile Delta of Egypt from September 2006 through September 2007. RNA was extracted from 10% fecal suspensions and reverse transcription PCR for astrovirus was performed as previously described (*4**–**6*). Astrovirus consensus primer pair SF0073/SF0076 amplified an ≈409-bp region of open reading frame (ORF) 1b, encoding the RNA polymerase gene. PCR-positive samples were then tested by using primer sets Mon269–Mon270 (*8*) and SF0053–SF0061, amplifying either a 449-bp or a 402-bp product of the ORF2 capsid gene from classical HAstVs (serotypes I–VIII) or astrovirus MLB1, respectively. DNA sequences of PCR products were determined by using Big Dye Terminator Cycle technology (Applied Biosystems, Foster City, CA, USA). Nucleotide sequences were compared with sequences obtained from GenBank. Phylogenies were constructed with the MEGA4 software (www.megasoftware.net) by using the neighbor-joining method and a p-distance algorithm. Bootstrap resampling was performed by using 2,000 replicates to demonstrate robustness of grouping. The nucleotide sequences determined in this study were assigned GenBank accession nos. HQ674630–HQ674650.

Consensus astrovirus reverse transcription PCR results were positive in 23 (6.3%) of 364 fecal samples. Five common serotypes of classical HAstV were identified constituting 16 (70%) of 23 positive samples; HAstV type I was most prevalent (n = 9). Alignment of the partial amino acid sequences of the ORF2 capsid region indicates that Egyptian HAstV type I strains share 99%–100% identity (Figure, panel A).

**Figure:**
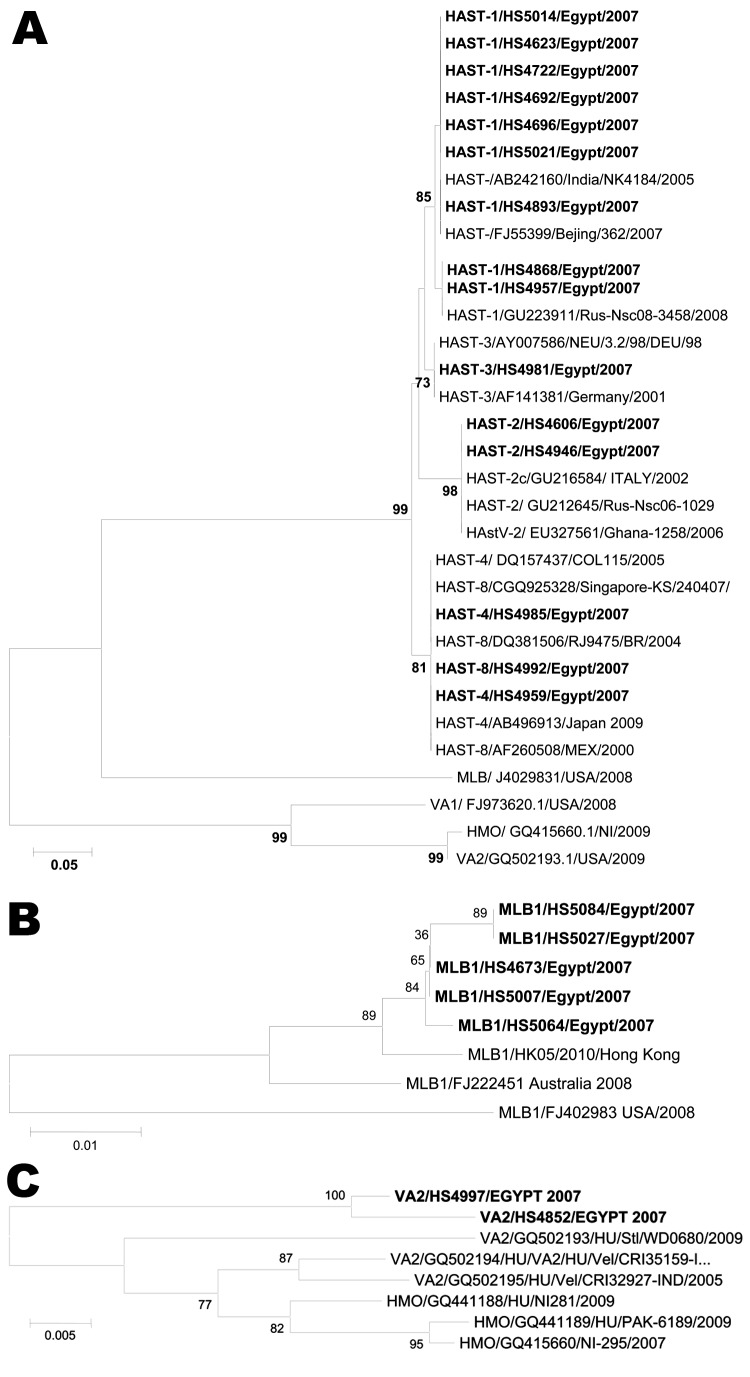
A) Phylogenetic analysis of the partial amino acid sequences of the open reading frame (ORF) 2 capsid region of human astrovirus (HAstV) types I–VIII (using Mon primer) with other sequences from GenBank. B, C) Phylogenetic trees based on partial nucleotide sequences of MLB1 ORF2 (B) and ORF1b (VA2) (C). Egyptian isolates are shown in **boldface**. Sequence alignment was performed by using ClustalW in the BioEdit software package (www.clustal.org). Dendrograms were constructed by using the neighbor-joining method as in MEGA4 (www.megasoftware.net). Values on the branches represent the bootstrap values from 2,000 replicates. Scale bar in A indicates amino acid substitutions per site; scale bars in B and C indicate nucleotide substitutions per site.

Five of the 7 remaining positive samples were most closely related to MLB1 on the basis of the partial ORF2 sequence analysis. Egypt MLB1 samples shared ≈99%-nt identity with each other, and all grouped in 1 phylogenetic cluster (Figure, panel B) along with a recently identified MLB1 strain (GenBank accession no. HM450380 [*9*]) isolated from a human fecal sample in Hong Kong. The Egypt MLB1 strains shared ≈97%-nt identity with the prototype MLB1 Australia strain but were more divergent from an isolate recently described in the United States (92% nt identity, GenBank accession no. FJ222451). However, all of the observed nucleotide differences represented silent mutations between Egypt MLB1 and Australia and US isolates; comparison of partial capsid protein sequences indicated no amino acid changes.

The 2 remaining HAstV-positive samples were phylogenetically most similar to astrovirus VA2 (VA2) (Figure, panel B). On the basis of the sequence of the amplicon from the ORF1b region, the Egyptian VA2 isolates shared 96.1%–100% aa identity to previously described VA2 and astrovirus human/mink/ovine genomes in GenBank.

Our study describes the detection of the recently identified viruses MLB1 and VA2 in a cohort of symptomatic children with diarrhea residing in Egypt. This study expands the geographic range of these viruses to include northern Africa. The consensus primers used in our study were able to detect a higher percentage of positive HAstV serotypes I–VIII than the Mon340/Astman-2 primers (*10*) (data not shown), a finding that encourages use of these primers to screen humans with gastroenteritis for astroviruses. Increased understanding of the genetic diversity within viral families infecting humans will assist in future studies of their pathogenicity and the design of specific diagnostic assays. Further epidemiologic studies, including clinical cases and demographically matched healthy controls, are required to better define their pathogenic potential.
